# Austin District Medical Society

**Published:** 1889-10

**Authors:** T. J. Bennett

**Affiliations:** Secretary


### SOCIETY NOTES.

### THE AUSTIN DISTRICT MEDICAL SOCIETY.

The eighth quarterly meeting of this Society was held in Austin on the 20th of September ultimo, with an attendance of thirty physicians.

Dr. Eugene Clark, of Lockhart, and Dr. C. O. Weller, of Austin, were admitted to membership.

Dr. J. W. Harrison read an interesting paper on

ANAL FISSURES, which was generally discussed.

Dr. A. N. Denton next read a paper on the much mooted question of

MATERNAL IMPRESSIONS, offering, perhaps, one of the best argunents in favor of this popular belief that has come out lately. The paper provoked quite a discussion. Drs. Denton, Daniel and Maxwell favored the doctrine, while Drs. Bennett, Swearingen and Tyner offered arguments against it. Suffice it to say other societies may now have the question; the Austin District has not settled it.

The next paper was a

REPORT OF A CASE OF PELVIC HÆMATOCELE, by Dr. E. Clark, read by Dr. F. E. Daniel, in the absence of Dr. Clark.

The subject that elicited the most discussion, and yielded the the greatest practical benefits, was

MALARIAL FEVERS AND TREATMENT, opened by Dr. T. D. Wooten, and participated in by nearly all present, particularly by Drs. Taylor, Swearingen, Daniel, Davis, Weller and F. Litten.

Dr. T. O. Maxwell made the following report of a case of

A WOMAN GORED BY A BULL.

The woman, when attacked by the bull, had a babe in her arms, and was carrying a pail of milk. The horns entered just above the pubis, and passed upward and to the left, coming out on the side a little above the superior spinous process of the ilium, making an ugly wound thirteen inches long. The abdominal cavity was opened, the wound through the peritoneum being three and a half inches in length. The bowels protruded.

The wound was semicircular in shape. The cavity was penetrated at the upper and convex border. The lower portion of the wound hung down as a flap.

The protruding bowels were thoroughly cleansed and placed back in the abdominal cavity. The wound was then cleaned off as well as possible, and stitched up with silk.

There was some suppuration at the outer angles of the main wound, but otherwise the patient made a satisfactory recovery.

The doctor stated that since fine stock had been introduced into his section of Travis county, four years ago, three women had been gored. One was killed outright, the animal's horn having penetrated the abdominal cavity and ranged upward, puncturing the diaphragm and wounding the heart.

Dr. Maxwell stated that it was generally held by stockmen that when a bull once gores a human being the animal would never do to trust afterwards, and should be killed.

The woman first mentioned was thrown entirely over the animal's back. The babe she held in her arms was not injured.

The Secretary's report for the two years of the Society's existence was read, and ordered spread upon the minutes. The report stated that the Society was organized with twenty-two members, and exactly doubled this number the first year, and the second year admitted seventeen, which now makes a total membership of sixty-one active, go-ahead physicians.

The report also stated that there were, within easy access of Austin, over two hundred regular physicians, besides some thirty odd irregulars of all grades.

The following officers were elected for the ensuing year:

Dr. F. R. Martin, of Kyle, President; Dr. T. J. Tyner, of Austin, First Vice-President; Dr. T. O. Maxwell, of Fiskville, Second Vice-President; Dr. T. J. Bennett, of Austin, re-elected Secretary and Treasurer for two years; Drs. S. E. Hudson, of Round Rock, and F. E. Daniel, of Austin, Censors, for one and two years respectively.

Dr. W. A. Morris will deliver his address as retiring President at the opening of the December meeting.

T. J. Bennett, Secretary.

